# Insight into the Surface Properties of Wood Fiber-Polymer Composites

**DOI:** 10.3390/polym13101535

**Published:** 2021-05-11

**Authors:** Klementina Pušnik Črešnar, Marko Bek, Thomas Luxbacher, Mihael Brunčko, Lidija Fras Zemljič

**Affiliations:** 1 Faculty of Mechanical Engineering, University of Maribor, 2000 Maribor, Slovenia; thomas.luxbacher@anton-paar.com (T.L.); mihael.bruncko@um.si (M.B.); 2 Faculty of Mechanical Engineering, University of Ljubljana, 1000 Ljubljana, Slovenia; Marko.Bek@fs.uni-lj.si; 3 Faculty of Chemistry and Chemical Technology, University of Maribor, 2000 Maribor, Slovenia; 4 Anton Paar GmbH, A-8054 Graz, Austria

**Keywords:** wood-fibers polypropylene composites, surface properties, skin-core structure, sustainable and biodegradable composites, ATR-FTIR, zeta potential measurement, nanoindentation, DPPH assay

## Abstract

The surface properties of wood fiber (WF) filled polymer composites depend on the filler loading and are closely related to the distribution and orientation in the polymer matrix. In this study, wood fibers (WF) were incorporated into thermoplastic composites based on non-recycled polypropylene (PP) and recycled (R-PP) composites by melt compounding and injection moulding. ATR-FTIR (attenuated total reflection Fourier transform infrared spectroscopy) measurements clearly showed the propagation of WF functional groups at the surface layer of WF-PP/WF-R-PP composites preferentially with WF loading up to 30%. Optical microscopy and nanoindentation method confirmed the alignment of thinner skin layer of WF-PP/WF-R-PP composites with increasing WF addition. The thickness of the skin layer was mainly influenced by the WF loading. The effect of the addition of WF on modulus and hardness, at least at 30 and 40 wt.%, varies for PP and R-PP matrix. On the other hand, surface zeta potential measurements show increased hydrophilicity with increasing amounts of WF. Moreover, WF in PP/R-PP matrix is also responsible for the antioxidant properties of these composites as measured by DPPH (2,2′-diphenyl-1-picrylhydrazyl) assay.

## 1. Introduction

The invention of the first plastics took place in the 19th century, and the development of new plastics continues. The material that overcame consumption in the last century is desired at all levels of our daily lives. To date, various types of plastics have been developed, divided into thermoplastics, thermosets, polyurethanes and elastomers [1,2]. The production of plastics increased from 2 million tons in 1950 to 380 million tons in 2015 and continues to grow worldwide [3,4,5]. The vast majority of these plastics are made from fossil hydrocarbons, are not biodegradable, and accumulate in the environment over time [4], with negative consequences for the entire planet. First, 5 to 13 million tons of plastics, or 1.5 to 4% of global plastic production, end up in the oceans every year. The environmentally damaging accumulation of plastics harms plants, animals, and industries such as tourism, fishing, and shipping. The production of plastics and the accumulation of plastic waste cause about 400 million tons of CO_2_ per year worldwide [1] Accordingly, there is a great trend to develop more biodegradable plastics. Plastic composites with added biodegradable components are most commonly formed and are now in vogue. It is important to add such components to the plastic that retain the useful properties of the plastic, such as mechanical properties, while increasing the level of biodegradability. Natural fibers as reinforced materials in polymer composites have attracted much attention due to their applicability in many fields such as automotive, aerospace, packaging, construction and transportation industries [6,7,8,9,10]. Natural fibers used as filler or reinforcing material in polymer composites, such as palm shells [11], palm leaves [12], guayule biomass, bagasse [13], sunflower stem flour [14], bananas [15], sugarcane, pineapple, ramie, and cotton [16], have many advantages, such as renewability, biodegradability, CO_2_ neutrality, non-toxicity, wide availability, low cost, low density, low energy consumption in manufacturing and high specific strength compared to synthetic fibers. Extremely important are also wood fibers (WF) and powders [17,18,19], while they are widely used and approved for the production of high performance wood-plastic composites [20,21,22]. Obviously, nowadays, the polymer composites reinforced with natural fibers are becoming increasingly important for the production of a wide range of engineering materials because they are relatively cheap, lightweight and environmentally friendly [23]. Most of the reviewed research has been devoted to the fabrication of natural fiber-thermoplastic polymer composites using chemically treated fibers or polymers, resulting in improved interaction between the wood and the thermoplastic material and consequently improved properties, such as (i) increased degree of crystallinity, (ii) mechanical properties, and (iii) rheological properties [24,25,26]. In contrast, our research focuses on the preparation of WF thermoplastic polymer composites with the same improved properties but without chemical pretreatment of the WF or incorporation of polymeric/inorganic additives. The production of such WF-polymer composites is referred to as “green production”, as sustainable and biodegradable materials are used that have a positive impact on the environment.

Although natural WF-polymer composites are very environmentally friendly, it is more difficult to achieve good adhesion/interaction of WF with the melting process of thermoplastic polymers during their production.

In our research, wood fibers (WF) were added to a polypropylene (PP) matrix to form “wood fiber composites” (WF-PP). Softwood was chosen as a filler, which was added to a PP matrix in different mass proportions (5%, 10%, 20%, 30% and 40%) during the extrusion process. In line with the increasing need to use recycled materials, we used both recycled PP and non-recycled PP (virgin) material as matrix material. In our previous work [27], these composites with different WF mass fractions were investigated for their chemical structure, thermomechanical and rheological properties and adhesion affinity. In this study, the surface properties were considered. It should be noted that most studies focus on the analysis of structural and mechanical properties and the state of research shows that surface analysis is underestimated for such materials. Surface properties such as wettability, morphology and surface chemistry as represented by composite water interface charge play a crucial role in the application of plastics. However, it is the surface, or the outermost layer of atoms of our solid that really defines how that material interacts with its environment and how it behaves for its intended purpose. These surface properties affect processes such as wetting, molecular adsorption, and interdiffusion, which determine how an interface turns into an interphase after the two materials are brought into contact. It should be noted that composites based on PP are quite often used as potential end products in such a way that their surface comes into contact with many different media (food, water, various solutions, biomasses), such as food plastics, containers, garbage cans, etc. [28,29]. These materials can also be used as packaging materials, among others, and the surface properties are the driving force for the effect of the plastic for which they are intended [30] and therefore it is important to study and understand them. In this paper, important surface parameters of WF-PP and WF-R-PP composites such as elemental composition, charge are discussed. Moreover, the nanoindentation method has been applied as an innovative approach to describe the mechanical properties of the surface of wood-thermoplastic-polymer composites to get an idea of the mechanical stability of the surface at the interface with other media. The DPPH (2,2′-diphenyl-1-picrylhydrazyl) spectrophotometric assay [31] was also performed to analyze the antiradical activity indicating the inhibition of oxidation processes, i.e., both the aging processes of the plastic itself and the inhibition of oxidation processes, e.g., in food packaging.

Our study confirms interesting findings from previous work that the untreated raw material WF acts as a nucleating agent that provides a new crystalline β-phase of PP, resulting in an increased degree of crystallinity of WF-PP composites, which may lead to improved specific final surface properties of the material. More so, the surfaces of WF-PP and WF-R-PP composites are associated with the WF loading and resulted in a skin-core structure and actually surpassed the antioxidant properties as well as the mechanical enhancement of the WF-PP composite. At higher concentrations of the WF, a smaller pure PP interface is found at the surface, i.e., decadent skin-core phenomena show up in higher relative modules and hardness. It has also been shown that the anionic nature of the composite (at the plateau level in the alkaline) decreases with increasing amount of added WF, which translates into a more hydrophilic character that directly affects the antioxidant activity of the surface.

## 2. Materials and Methods

### 2.1. Materials

Unrecycled PP, AMPPLEO 1020 GA (0.94 g/cm^3^, 20 g/10 min: MFI) (Philadelphia, PA, USA) was purchased from Braskem, and recycled PP Eco Meplen IC M20 BK (0.90 g/cm^3^, 50 g/10 min: MFI) was purchased from MEPOL S.r.l. (Treviso, Italy), Wood Fiber was obtained locally (softwood). Ultrapure water was dispensed by a MilliQ water purification unit (Millipore Corporation, Danvers, MA, USA). Electrolyte solutions for the surface Zeta Potential analysis were prepared from KCl p.a., 0.1 moL/L HCl and 0.1 moL/L KOH, purchased from Carl Roth, Germany.

### 2.2. Synthesis Methods

#### 2.2.1. PP and Wood Fiber

As we reported in our previous work [27] for the preparation of wood fiber-reinforced composite PP materials, we used in this research two different matrix materials (i) Polypropylene (PP) from Braskem and (ii) Recycled Polypropylene (R-PP) material obtained from MEPOL. Both matrix materials were reinforced with wood fibers obtained from the wood processing company (MLINAR d.o.o.) as a side product of plywood grinding. The wood fibers consisted of spruce and pine wood (roughly −20–80%).

#### 2.2.2. Wood-PP Composite Material Preparation

Wood PP/R-PP composites were prepared by melt compounding WF and PP/R-PP without adding processing additives.

Prior to extrusion, WF and polypropylene were dried at 90 °C for 2 h in a ventilated oven. WF-PP and R-PP were mixed and fed to the feed unit of the twin screw extruder, PolyLab HAAKE Rheomex PTW 16, Thermo Haake. To ensure uniform distribution of fibers in the polymer matrix, all materials were extruded four times. After each extrusion, the material was pelletized by a pelletizer, and the material was again fed to the feeder. WF-PP and WF-R-PP composites were prepared with different mass fractions of WF, ranging from pure polymer (matrix) material to a maximum wood content of 40 wt%. In total, six materials with PP and six materials with recycled PP were produced. An overview of the materials produced is shown in Table 1, with names given to the individual material blends.

A melt temperature of approximately 190 °C was used for all materials and all four extrusion cycles, which required adjusting the temperature along the length of the extruder barrel depending on the material extruded. In all cases, the screw speed was set to 80–90 rpm and the feed stage to 70–80.

**Figure 1:**
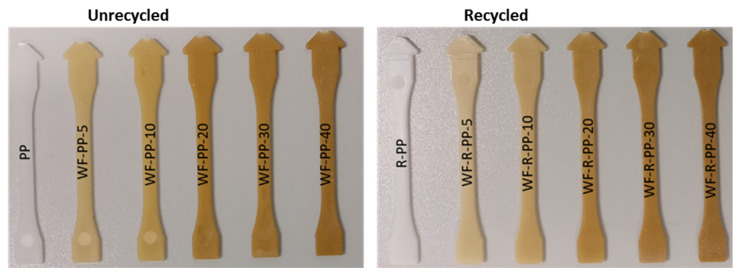
Samples composites with different wt. % of WF for unrecycled and recycled PP composite materials.

The dumbbell shaped samples were prepared following the ISO 3167:2014 Standard (sample type 1B). Prior to the injection moulding, the material, in granulated form, was dried at 80 °C. Dumbbell samples were prepared using the injection moulding machine, Arbrug Germany, with 15 t of closing force. For the injection phase, a pressure between 700−900 bar and 190 °C melt temperature was used, while the temperature of the mould was set to 20 °C. The injection step was followed by backpressure steps of 500 bar and 100 bar. The last step was cooling in the mould for 12 s. The whole production cycle for 1 sample lasted for 18 s. Figure 1 shows the injection moulded samples of each material.

**Figure 2:**
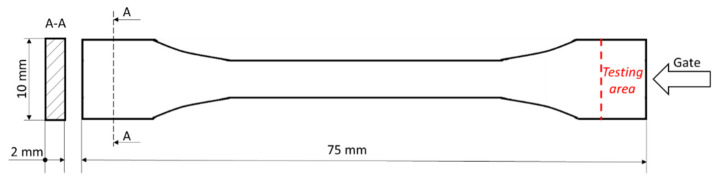
The testing area for surface analyses of dumbbell shaped sample.

As specified, surface tests were made on the dumbbell shaped samples seen in Figure 2. All surface tests were made in an area closer to the injection gate, indicated with a dotted line in Figure 2.

The samples of composites WF-PP/WF-R-PP, which were used for an antiradical assay, were prepared using a custom-made spherical cylinder. This spherical cylinder, with a size of 12 mm radius and 2.5 mm height, was processed under the same conditions as the dumbbell-shaped samples and using the injection moulding machine Arbrug Germany with 15 t clamping force (a pressure between 700−900 bar and 190 °C melt temperature was used, with the mould temperature set at 20 °C; the injection step was followed by backpressure steps of 500 bar and 100 bar, and the last step was cooling in the mould for 12 s).

### 2.3. Analytical Methods

#### 2.3.1. Particle Size of Wood Fibers

The volume size and size distribution of WF were characterized in our previously work [27] by laser diffraction using a Particle Size Analyzer (PSA 1190), Anton Paar, Austria. To measure the WF properties, a WF-water mixture was first prepared. This mixture was first sonicated for 30 s to break up any agglomerates. The mixture was then measured for 10 s. Seven replicates were performed, and the average size distribution was determined.

#### 2.3.2. Dumbbell Shaped Samples

**X-ray Diffraction Analysis**

X-ray diffraction (XRD) measurements, by a PANalytical PRO MPD diffractometer using a Cu Kα radiation source at 40 kV were used for determination of the crystalline structure of WF-PP/WF-R-PP composites The X-ray diffraction patterns were recorded for the angles in the range of 2 Ɵ, from 10 to 40°, with a step of 10°/min (λ = 0.154 nm).

**ATR-FTIR Spectroscopy**

To provide detailed information of the surface chemical structure of WF-PP and WF-R-PP composites provided by the extrusion and injection moulding process, the ATR-FTIR spectra were monitored on a Perkin Elmer Spectrum GX NIR FT-Raman. (The analyzed samples of the surface were in contact with a diamond crystal.) Each spectrum was determined as an average of 32 scans at a resolution of 4 cm^−1^ for measuring background, and the samples’ spectra were measured in the wavenumber range from 400 to 4000 cm^−1^ at room temperature. All spectra were baseline corrected and smoothed after the measurements. An approach was used to estimate the changes in the C-O groups in the composites WF-PP/WF-R-PP. Thus, those of the individual spectra were base corrected from 5 to 40% of WF addition and the area was mathematically calculated using appropriate mathematical models (Gaussian terms).

**Microstructure Characterization**

The microstructure of the injection moulded samples was examined on a transversal cross-section with an optical microscope, Nikon Epiphot 300 (Tokyo, Japan), equipped with a system for digital quantitative image analysis (Olympus DB12 and software program Analysis). Before metallographic preparation, the samples were positioned using metal clamps and cold mounted carefully in epoxy resin. Grinding was performed using SiC paper P400 followed by polishing using a 9- and 3-micron diamond suspensions, and in the final step 0.05-micron colloidal alumina. In all polishing steps a micro-cloth was used, which was wetted prior to applying the polishing agent for additional lubrication. Samples were prepared on an automated grinder/polisher, using clockwise rotation 150/40 rpm, and a force of 27 N.

**Zeta Potential Measurements**

For the surface Zeta Potential analysis with SurPASS-3, two sample pieces of approx. 10 mm × 9 mm were cut for the streaming potential measurement, as indicated by the dotted lines in Figure 2. The sample pieces were fixed on the sample holders of the Adjustable Gap Cell (with a cross-section of 20 mm × 10 mm) using double-sided adhesive tape. The sample pieces were aligned opposite of each other, such that the maximum surface area of the samples overlapped. The distance between the sample pieces was adjusted to 105 ± 5 µm. Streaming potential measurements were performed using an aqueous 0.001 moL/L KCl solution as the background electrolyte. The pH dependence of the surface Zeta Potential was determined by adjusting an initial pH 10, using 0.05 moL/L KOH and reducing the pH automatically by dosing 0.05 moL/L HCl.

**Nanoindentation**

Nanoindentation measurements of elastic modulus and hardness were performed using a G200 Nanoindenter (Agilent, Santa Clara, CA, USA) equipped with an XP head and Berkovich geometry (indenter tip). Continuous Stiffness Measurements (CSM) methodology was used to obtain properties throughout the selected indentation depth [19]. A harmonically oscillating tip with frequency 45 Hz and 2 nm amplitude was pushed into the material. Based on the dynamic model of the whole measuring system, the sample properties were calculated continuously up to the penetration depth of 3900 nm. Measurements were performed on the sample surface (Figure 2) with 36 (6 × 6) indents per sample. The distance between indents was 150 µm. For the analysis, all measurements were averaged.

**The DPPH° Assay**

**Figure 3:**
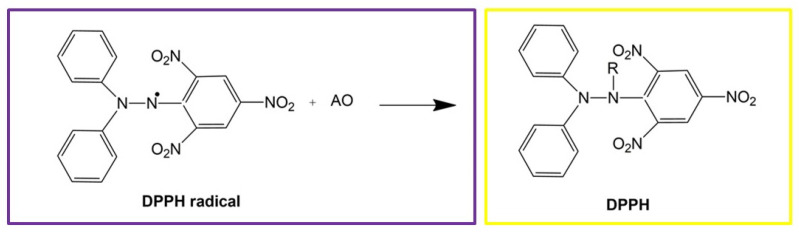
DPPH. Free radical reaction with an antioxidant (AO).

The antioxidant activity of WF-PP and WF-R-PP composites was measured using DPPH• (2,2′-diphenyl-1-picrylhydrazyl) (Sigma Aldrich, France). The method is established on the reduction of the DPPH• radical, which is analyzed spectrophotometrically at a wavelength of 515 nm (Spectrophotometer (UV-VIS) Agilent Cary 60) (Figure 3).

DPPH• organic radical can be reduced in the antioxidant (AO) presence, with the consequent decolorization, from purple to yellow color. The antioxidant capacity can be determined by decrease of absorption at wavelength 515 nm. DPPH solution was prepared in methanol (8.1 × 10 − 5 moL/L) [32,33]. The WF-PP/WF-R-PP composites samples disks were directly immersed into 3 mL of the methanol DPPH• solution. The scavenging capability was determined straightaway, after 100 min, 200 min and 300 min. The percentage of radical scavenging activity at 515 nm was calculated using Equation (1):

(1) $$Inhibition\;=\;\left(A_{\mathrm{Control}}-A_{\mathrm{Sample}}\right)/A_{\mathrm{Control}}\cdot \;100\%\;$$

where *A*_Control_ is the absorbance, measured at the starting concentration of DPPH•, and *A*_Sample_ is the absorbance of the remaining concentration of DPPH• in the presence of WF-PP and WF-R-PP composites polymer.

## 3. Results

### 3.1. Wood Fibres

In our previous work [27] it was published that the average size of WF as fillers was about 100 μm. Few WF had sizes smaller than 10 μm, and, on the other hand, the diameter of some of the largest particles was about 500 μm.

### 3.2. Surface Properties of Injection Moulded Samples of Wood Fibre-Polypropylene Composites

**Figure 4:**
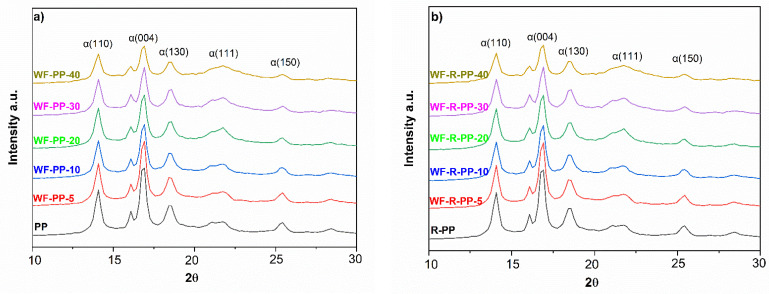
XRD patterns of (**a**) PP, WF-PP composites and (**b**) R-PP, WF-R-PP composites.

X-ray diffraction patterns show crystallization of PP/R-PP in the presence of WF (Figure 4a,b). The diffraction peaks for PP shown in Figure 4a are positioned at 2 θ angles of 13.9° (110), 16.8° (004), 18.5° (130), 21.4° (111) and 28.6 (200). All these peaks reflect the α-crystalline phase of PP. The diffraction peaks of PP based WF composites were determined at 2θ angles of 13.9° (110), 16.8° (004), 18.5° (130), 21.4° (111) and 28.6 (200). On the other hand, the diffractograms of R-PP and all WF-R-PP composites were also evaluated in Figure 4b. The results represented that there is no difference in the diffraction peaks. The diffraction peaks of R-PP-WF composites known for the α-crystalline phase were determined at 2θ angles of 13.9° (110), 16.8° (004), 18.5° (130), 21.4° (111) and 28.6 (200), respectively. The results of PP and R-PP polymer matrix based WF composites show that there is no difference in the diffraction peaks, and there is no evidence of phase transformation. In our previous study [27], XRD measurements reveal the changed crystallographic structure of PP/R-PP. Even more, it was clarified that the presence of WF with nucleation ability leads to the performance of formation of a different β-form and γ-form phases in PP. In contrast, in the following study, all WF-PP/WF-R-PP composite samples preferentially followed the α-monoclinic PP formation.

**Figure 5:**
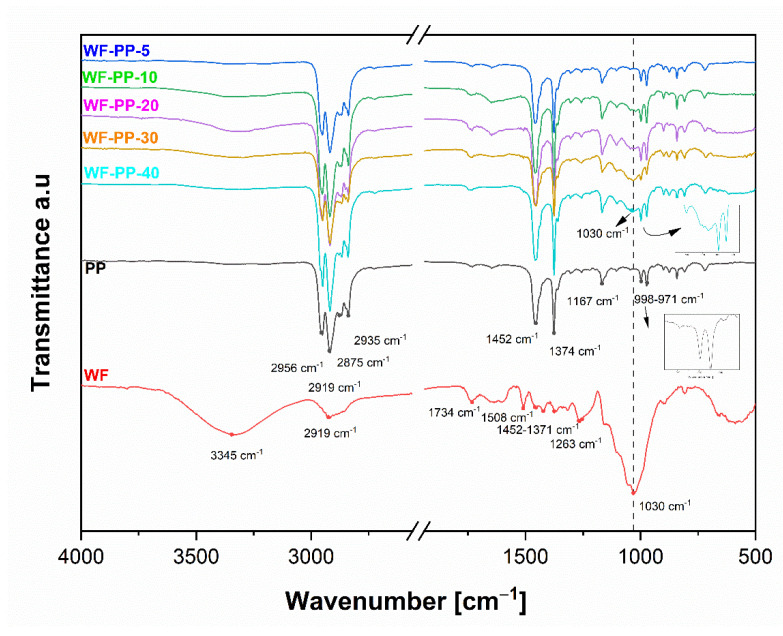
ATR-FTIR spectra of unrecycled PP, WF and wood fiber-polypropylene composites (WF-PP) injected moulded samples with different addition of WF content (from 5%:WF-PP-5 to 40%:WF-PP-40).

The elemental composition of the composite surfaces was monitored by ATR-FTIR analysis. The spectra of the injection moulded samples with different addition of WF (from 5%: WF—PP−5 to 40%: WF—PP−40) as well as the pure PP and WF, are shown in Figure 5. As can be seen from this Figure 5, the most prominent characteristic fingerprint band vibration at 1030 cm^−1^ (associated with the stretching vibrations of different groups in carbohydrates) appeared in the sample with the highest proportion of WF, i.e., 40%. Figure 5 shows the ATR-FTIR spectra of unrecycled PP (PP), wood fibre (WF) and wood fibre-polypropylene composites. The characteristic bands of PP (Figure 5) and R-PP (Figure 5) at 2919 cm^−1^ correspond to the asymmetric CH_2_ stretching vibration, the band at 2956 cm^−1^ to the -CH_3_ stretching vibration and 2875 cm^−1^ to the asymmetric -CH_2_ stretching vibration. The characteristic peaks at 1452 cm^−1^ and 1374 cm^−1^ were determined as -CH_3_ -CH_2_-rocking vibration. In the “fingerprint” region of PP and R-PP, the spectra contain several main PP tacticity, which includes isotactic, syndiotactic and atactic forms. Typical characteristic peaks for isotactic PP are found in the region below 1000 cm^−1^ [34,35]. The characteristic vibration bands of WF (red curve) are also shown in Figure 5. WF are composed of cellulose, hemicellulose and lignin [36,37]. Since the wood structure is very complex, the ATR-FTIR spectra were divided into two regions. The first region from 3800 cm^−1^ to 2700 cm^−1^ includes the OH and CH stretching vibrations. A strong broad peak is seen at 3345 cm^−1^, which has been assigned to various O-H stretching vibrations, while at 2919 cm^−1^ a characteristic peak is related to the asymmetric and symmetric methyl and methylene stretching vibrations groups represented in wood. The second, at 1800 cm^−1^ to 800 cm^−1^, is known as the “fingerprint” region for various functional groups of the wood structure. The “fingerprint” region of wood consists of several bands related to wood structure. A characteristic peak associated with the C=O stretching vibration in lignin and hemicellulose was observed at 1734 cm^−1^. The characteristic bands at 1508 cm^−1^ and 1263 cm^−1^ were determined to be C=C, C-O stretching or bending vibrations of the groups in lignin. The bands at 1452 cm^−1^, 1421 cm^−1^, 1371 cm^−1^ were assigned to C-H, C-O deformation, bending or stretching vibrations of lignin groups and carbohydrates. The bands at 1023–1051 cm^−1^ were assigned to C-O deformation in cellulose, symmetric C-O-C stretching of dialkyl ethers and aromatic C-H deformation in lignin [38,39] generally for carbohydrates from WF.

**Figure 6:**
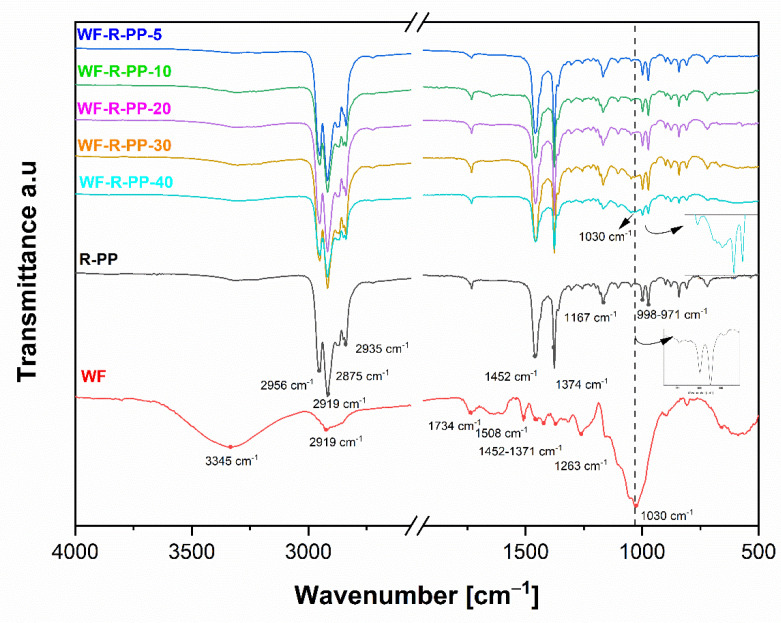
ATR-FTIR spectra of recycled PP, WF and wood fibre-polypropylene composites (WF-R-PP) injected moulded samples with different additions of WF content (from 5%:WF-R-PP-5 to 40%:WF-R-PP-40).

We see that there are no significant differences between the pure PP material and the composites up to a concentration of 30% by weight of added WF. Since we measure the properties at the surface, this indicates that a layer of (only) PP has formed at the surface of the sample. At the highest concentration (30, 40%), characteristic band oscillations occur at 1030 cm^−1^. This indicates C=O, C-H, C-O-C, C-O deformations or stretching vibrations of the different functional groups in carbohydrates with WF origin. A similar behavior in the surface chemical composition was observed for the orientation molecules on the surface of the injection moulded samples of the WF-R-PP recycled-based composites PP. Figure 6 shows the results for the recycled composites, WF and R-PP. The surfaces of WF-R-PP composites with lower additions of WF show no significant difference in the characteristic bands, and so it could be suggested that only R-PP is oriented in the upper layer of the material. Only the injection moulded samples of WF-R-PP composites with the highest WF content (i.e., 30, 40%) showed characteristic band vibrations at 1030 cm^−1^ indicating C=O, C-H, C-O-C, C-O deformation or stretching vibrations of the different functional groups in the carbohydrates of the WF origin.

**Figure 7:**
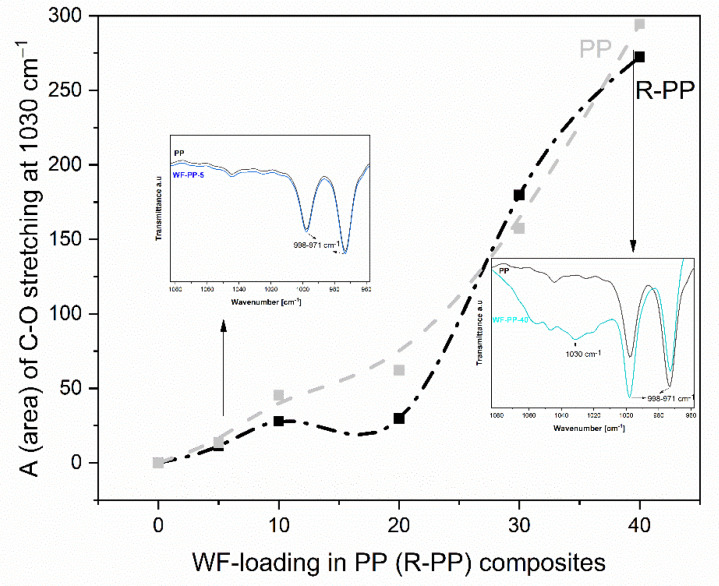
The fraction area (A) of C-O stretching groups at 1030 cm^−1^ in WF-PP and WF-R-PP composites as a function of loading. WF.

We have also critically analyzed the ATR-FTIR data at wavelength cm^−1^ by fitting mathematical models (Gaussian terms) to find out the orientation of the functional groups WF on the surface of the composites WF-PP and WF-R-PP. Figure 7 shows the dependence of the area of the peak at 1030 cm^−1^ representing the stretching vibration of the C-O and C-O-C groups of the composites WF-PP and WF-R-PP with respect to the functional groups WF. Spectral analysis had shown that significant spectral differences occur at the surface of WF-PP and WF-R-PP. The characteristic peak at 1030 cm^−1^ increased with increasing WF loading in WF-PP/WF-R-PP composites. The results for A (area) were plotted as a function of WF loading in WF-PP and WF-R-PP composites. Based on this fit, there appears to be a broadening of this peak in cases up to 30% of the addition of WF to PP or R-PP. This indicates the increasing presence of WF functional groups on the surface of WF-PP and WF-R-PP composites. We also detected the signals of these bound contributions, as indicated in Figure 5 and Figure 6, which are much stronger in WF-PP-30, WF-PP-40, WF-R-PP-30, WF-R-PP-40 compared to PP or R-PP.

Comparison of the two ATR-FTIR spectra of WF-PP and WF-R-PP composite samples at the surface layer indicates the same behavior of PP -orientation in WF-PP and WF-R-PP composites. PP was oriented by injection moulding process, preferably at the surface. This process is known from the literature and is defined as skin-core orientation of PP polymer [28,29,40,41,42,43,44,45]. In the literature, the relationship between the thickness of the polymer surface skin layer and the injection speed and temperature has been clearly studied. At a lower speed, the thickness of the skin is higher because the melt front of the material has more time to relax and is more prone to cooling. At lower mold or melt temperatures, the opposite is true. The skin layer increases. At the highest injection rate, the mold and melt temperatures have a negligible effect on the skin thickness [46].

In the skin-core process, the material enters in a parabolic profile, taking into account the stretching and deposition on the wall and the formation of an immobile freezing layer. The top layer of molten material flows within the shell, and the thin region of the extended layer is called the skin. The core region, on the other hand, solidifies last and relaxes as it cools. The hierarchical structure is therefore commonly referred to as the skin-core structure and, following thermoplastic composites, involves a phase behavior for the dispersed phase and a crystalline or oriented structure hierarchy for the matrix.

Only in 30%, 40% WF addition, some of these fibers are obviously oriented and present on the composite surface, which is due to the identification of the functional groups present in the wood (cellulose, hemicellulose as carbohydrates and, lignin.).

**Figure 8:**
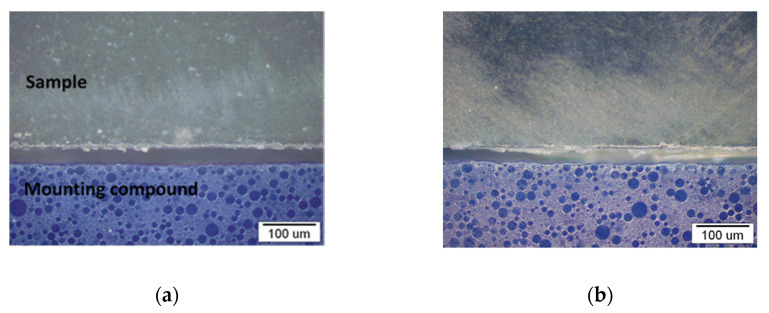
Micrographs of the boundary surfaces of the samples: PP (**a**), R-PP (**b**).

**Figure 9:**
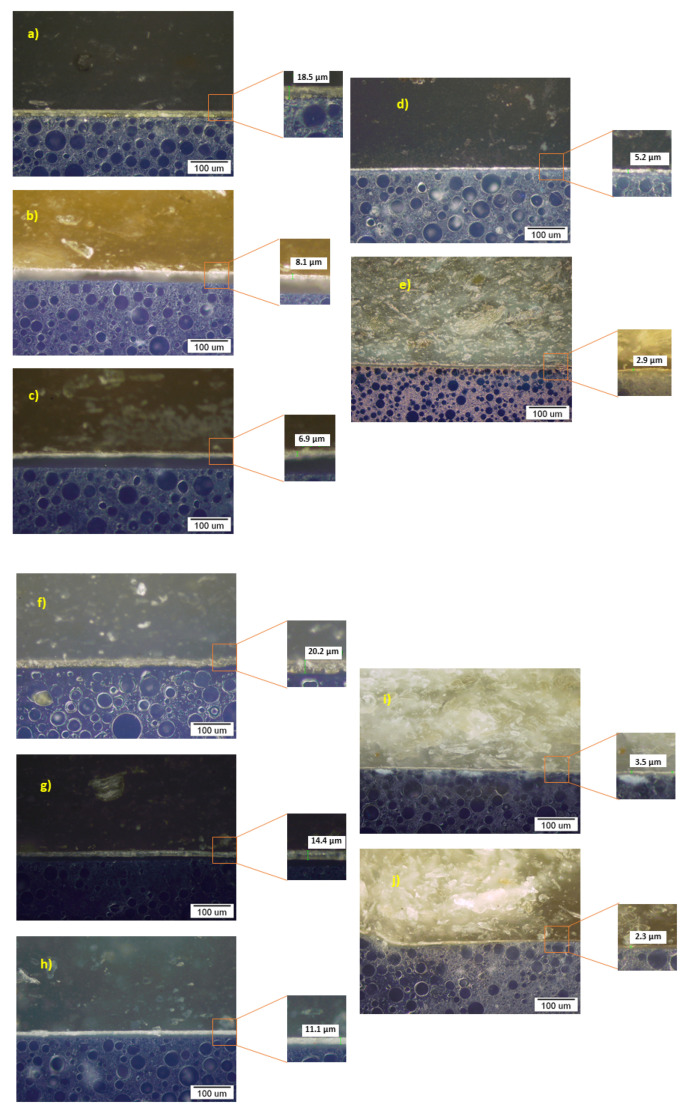
Micrographs of the boundary surfaces of the samples: WF-PP-5 (**a**), WF-PP-10 (**b**), WF-PP-20 (**c**), WF-PP-30 (**d**), WF-PP-40 (**e**) and WF-R-PP-5 (**f**), WF-R-PP-10 (**g**), R-WF-R-PP-20 (**h**), WF-R-PP-30 (**i**) and WF-R-PP-40 (**j**); cross section, polished state, optical microscope, dark field.

The relationship between WF elongation on PP/R-PP with the increased distribution of skin phenomenon was further confirmed by the light microscopy images (Figure 8, Figure 9). The main objective of this study was to determine the microstructure of WF-PP/WF -R-PP composites from the skin core region and to investigate the evolution of the structure from the practical point of view of these WF-PP composites. The skin layer is known for its strong orientation with the flow. It has been shown in the micrographs of the interfaces of PP/R-PP and WF-PP/WF-R-PP composites.

As can be seen for the same injection pressure and temperature as PP/R-PP and all WF-PP/WF-R-PP specimens, the thickness of the boundary layers is mainly affected by the WF loading. Moreover, the thickness of the skin layer represented on Figure 9 varied from 18.5 μm for WF -PP-5 composites to 2.9 μm for WF -PP-40. There was not much difference in the thickness of the skin layer, about 7.8 μm, between the composites with WF loading from 10 to 20%. In fact, the recycled WF-R-PP composites showed the same trend of skin layer thickness, from 20 μm for WF-R-PP−5 composites to 2.3 μm for WF-R-PP−40 composites. Compared to PP/R-PP, the layer is thicker and varies from 28.3 to 34.7 μm. The reason for the decreasing effect of skin thickness is probably due to the orientation of WF in the PP matrix, which are preferentially oriented in the core layer with PP matrix at low WF loading. Since this has been clearly studied in the case of PP-PET microfibril composites [45], we can predict the same conclusions there as well. When the content of WF in PP is low, the PP melt pushes WF into the mold, resulting in a preferential orientation of WF in the core. When the loading of WF in the PP matrix increases, the orientation of WF in the thin skin layer is also oriented. The results are in agreement with literature data, and it was found that with increasing WF loading, the skin layer of the polymer (PP, R-PP) becomes degreased, and this is more pronounced at 30 and 40% added WF. The latter is in accordance with the ATR-FTIR results.

**Figure 10:**
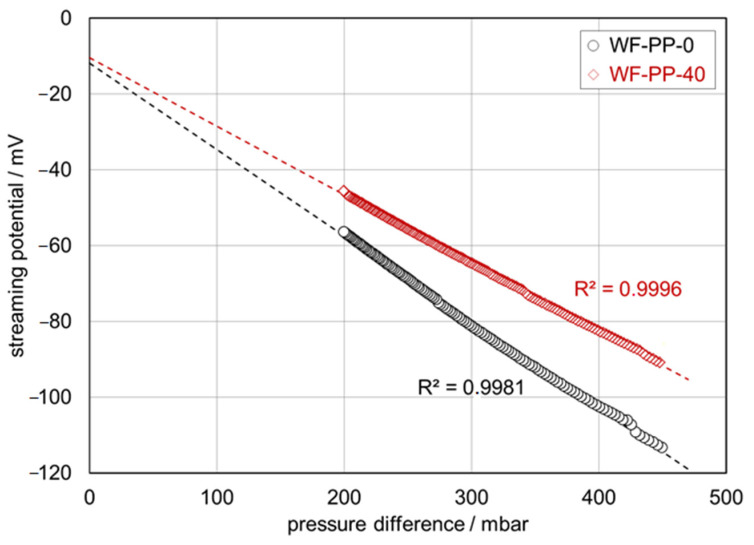
Pressure ramps (streaming potential vs. pressure difference) for WF-PP-0 and WF-PP-40 in 0.001 moL/L KCl at pH 6.1.

Among the surface parameters, the surface charge is a key parameter for enhancing or suppressing the interaction between dissolved compounds in an aqueous solution and solid material surfaces [47]. The zeta potential is a representative for the surface charge at the solid-water interface and a valuable parameter for the comparison of material surfaces before and after surface modification. The zeta potential is also applicable to characterize the effect of blending PP matrix with softwood fibers provided that the bulk composition of the WF-PP composites is reflected at the surface. Zeta potential results were thus obtained for unrecycled polypropylene (PP), recycled polypropylene (R-PP), and PP and R-PP with embedded softwood fibers (5−40 wt%). Figure 10 compares the raw measuring data, which is the dependence of the streaming potential on pressure difference, for pure PP and WF-PP-40 composite made of pure PP and 40 wt% WF at pH 6.1. We see a clear difference in the (negative) slope of the linear dependence of streaming potential on pressure difference, i.e., the streaming potential coupling coefficient dU_str_/dΔp, which is then used to calculate the surface zeta potential [48]. At pH 6.1 we obtain ζ = −55.1 mV for PP and ζ= −39.5 mV for WF-PP 40.

**Figure 11:**
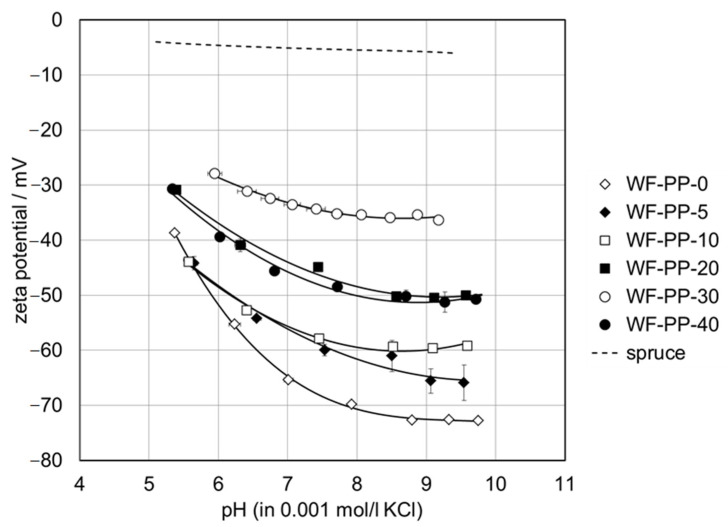
Zeta potential of WF-PP composite samples in the range of pH 5−10. The evolution of the zeta potential with pH for spruce as a representative softwood is shown for comparison.

Figure 11 shows the zeta potential of PP with different addition of softwood fibers (5−40 wt%) as a function of pH in the interval of pH 5−10. The extension of the pH dependence of the zeta potential towards lower pH approaches the isoelectric point (i.e., p.) in the range of pH 3−4 (data not shown). The sensitivity of the i.e., p.s of pristine and scarcely functionalized polymer surfaces towards traces of impurities on the polymer surface and sample pre-treatment protocols make this parameter less applicable for a distinction between pristine PP and WF-reinforced composites. Instead we focus on the higher pH range where the zeta potential assumes a steady value due to the saturation of the polymer surface with hydroxide ions [49] or the complete de-protonation of acidic groups of WF exposed at the composite-water interface. For a series of alike material surfaces with a varying number of surface functional groups, the zeta potential correlates with surface hydrophilicity, which is usually represented by the water contact angle [50]. Such correlation is best observed when using zeta potential results obtained at higher pH, e.g., pH 8−9.

**Figure 12:**
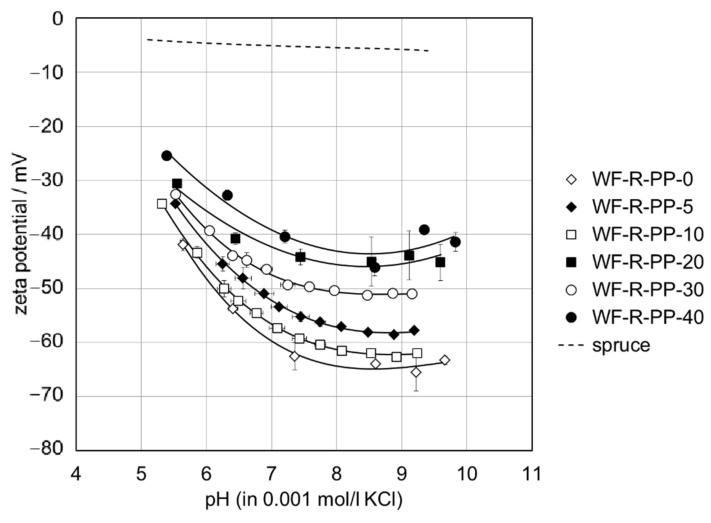
Zeta potential of WF-R-PP composite samples in the range of pH 5−10. The evolution of the zeta potential with pH for spruce as a representative softwood is shown for comparison.

From Figure 11 it can be seen that the contribution of the softwood fiber surface to the overall zeta potential of the WF-PP composites causes the shift of the negative zeta potential at high pH towards more positive values. As a representative indicator for the effect of the bulk WF fraction on the surface and interfacial charge, the negative zeta potential obtained at pH 8 is given in Table 2 for the series of WF-PP composites. It becomes evident that the zeta potential at pH 8 does not describe a continuous trend but reflects the discrepancy between bulk and surface compositions of the WF-PP composites. When comparing the experimental results for the zeta potential of WF-PP composites at pH 8 with the predicted zeta potential calculated by the weighted average of the zeta potential for unrecycled PP and spruce we conclude that except for WF-PP-40 the zeta potential of WF-PP composites suggests a higher surface concentration of softwood fibers compared to their bulk composition. In general, it may be seen that with increasing the added WF the ζ-potential at pH 8 became less negative. With the addition of WF to both PP and recycled PP composites (Figure 11 and Figure 12), polar groups are introduced onto the composite surfaces (mainly OH and COOH), which cause the increase of surface hydrophilicity and thus a further decrease in the magnitude of the zeta potential at high pH. Figure 11 and Figure 12 shows the corresponding zeta potential results for recycled PP and for R-PP with 5−40 wt% softwood fibers again in the range of pH 5−10.

Changes in the zeta potential are already evident with small additions of WF, which is probably also due to the analysis in the wet, where, in addition to the chemical nature, a faster wettability and reflection of the hydrophilic or hydrophobic character of surfaces occurs also due to morphological changes.

**Figure 13:**
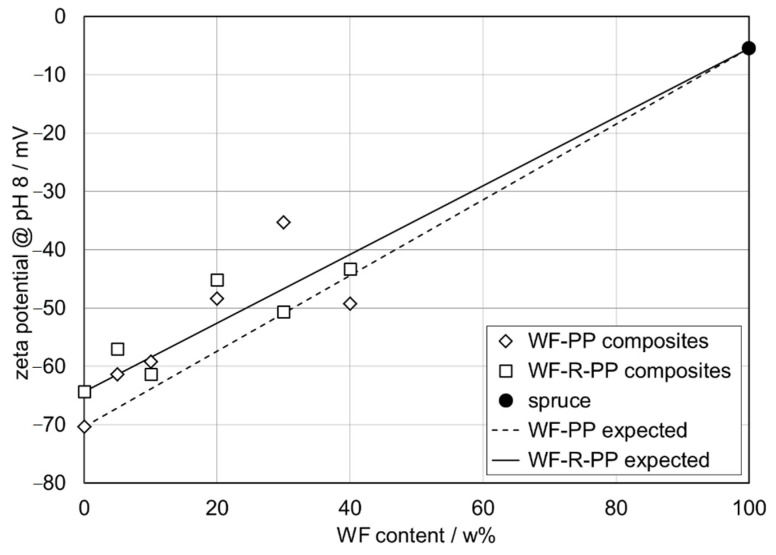
Correlation between the plateau zeta potential at pH 8 and the contents of softwood fibers in PP- and recycled PP-wood fiber composites.

A comparison of the zeta potential for the series of WF-PP and WF-R-PP (Figure 13) composites generally shows that recycling of PP shifts the zeta potential at high pH to slightly less negative values. The small difference in the surface hydrophilicity between the unrecycled and recycled PP resins of the PP-wood particle composite samples is evident, which may be related to the contribution of polyethylene (PE) to R-PP. For the WF-PP composites the difference in hydrophilicity for the neat polymers PP and R-PP is superimposed on their surface properties and thus reflected by the composites’ zeta potential.

By taking a closer look at the deviation of the measured zeta potential for the WF-R-PP composites from the predicted zeta potential derived from the zeta potential of the unrecycled materials R-PP and spruce we realize that the zeta potential results for WF-R-PP resemble the expected trend much better than the zeta potential for WF-PP. We conclude that the softwood fibers show a stronger affinity toward recycled PP while they are less accepted by the more hydrophobic pristine PP resin. For the latter composite softwood fibers experience a stronger repulsion in the bulk and tend to accumulate in the proximity of the surface.

An important conclusion of the zeta potential measurements is that in all composites the anionic charge dominates in almost the whole pH range. This clearly indicates that these plastics have a high affinity for adhesion/adsorption of cationic substances and repeal anionic substances. In this way, some adsorption affinity and electrostatic interactions of those composites may be predicted.

The further investigation was focused on nanoindentation measurements. As already mentioned, many such materials act on the interphase, so the first mechanical loads occur on their surface, it is highly recommended to monitor the mechanical properties of the surface layer of the material.

**Figure 14:**
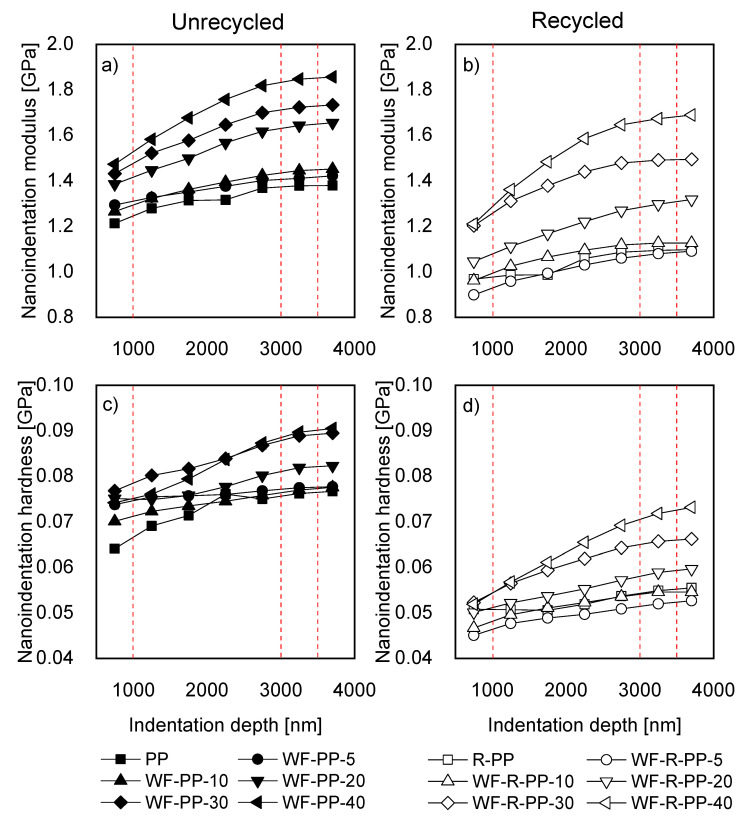
Nanoindentation modulus as a function of indentation depth for (**a**) unrecycled and (**b**) recycled materials. Nanoindentation hardness as a function of indentation depth for (**c**) unrecycled and (**d)** recycled materials.

The composite materials were analyzed by nanoindentation measurements, where the materials were tested to a maximal depth of 3900 nm. This range was subdivided into intervals of 500 nm, starting from 500–1000 nm, and going to a final interval of 3500–3900 nm. Within each interval, the nanoindentation modulus and hardness were averaged. The results of this analysis are shown in Figure 14. The red dotted lines in Figure 14 indicate the two indentation depths that were analyzed in detail in continuation (Figure 14).

The modulus of both materials, regardless of the wood concentration, increased with indentation depth. The rate of change decreased with increasing wood concentration and plateaued at around 3000–3500 nm depth. The increasing modulus with indentation depth could result from a higher concentration of WF through the sample depth, different polymer matrix structures, or a consequence of the substrate effect, where the bottom layers affect the properties of the top layer.

Results suggest that, at the surface layers (up to 1000 nm), where we see minor differences between the materials and smaller modulus values, the pure polymer matrix material is present primarily. The increasing modulus with indentation depth could result from a higher concentration of WF through the sample depth, different polymer matrix crystalline structure caused by the injection moulding, or a consequence of substrate effect where the bottom layers affect the properties of the top layer. From Figure 14a the influence of WF’ concentration on modulus can be seen for unrecycled material. However, similar conclusions can be drawn for recycled material, Figure 14b. The materials with 5% and 10% wood concentration (WF-PP-5 and WF-PP-10) follow the behavior of pure polymer material (PP). For clarity, we did not show the error bars on these diagrams. However, the error bars of materials with 0, 5, and 10% of WF were overlapping, indicating that there was practically no difference between these three materials. The skin core effect can explain similar mechanical properties. As observed by optical microscopy and confirmed by ATR-FTIR, the materials form a thicker PP layer in the case of materials with a lower concentration of WF. Measurements show that in the surface layers (up to 1000 nm), where we see marginal differences between the three materials, the pure polymer matrix material is present primarily.

From 20 wt. % of WF on, higher modulus values compared to the previous three concentrations can be observed. A higher modulus is seen at all indentation depths. The increase of the modulus can be attributed to decreasing layer of pure PP. It was shown from optical microscopy that at 20 wt. % the PP layer is 6900 nm thick for PP-based composites and 11,000 nm thick for R-PP-based composites. This layer decreases to 2900 nm (PP composites) and 2300 nm (R-PP composites). As the layer decreases, the substrate effect becomes more pronounced. And in the case of 30 and 40% wt. materials, the PP layer is thinner compared to indentation depth.

The comparison of the modulus values of unrecycled (Figure 14a) and recycled (Figure 14b) materials show that recycled materials have lower values of modulus regardless of the WF concentration. Comparing the pure polymer matrix materials (PP and R-PP) shows an inherent difference between the two matrix materials. Recycled material (R-PP) has modulus values lower by about 0.25–0.30 GPa throughout the surface depth. The lower modulus of pure matrix material may result from the recycling process since, during this process, the polymeric material is exposed to harsh conditions that could lead to material degradation. Another reason for lower modulus could be the presence of polyethylene-origin material that DSC detected in the R-PP material in our previous work [27]. Generally, PE materials have lower modulus values compared to PP materials. Therefore, a combination of PP and PE could result in a lower modulus compared to PP material. R-PP material’s lower modulus also influences the mechanical properties of filled materials, as the modulus of all recycled filled materials is lower compared to unrecycled materials.

The results of hardness (Figure 14c,d) showed the same trends as in the case of modulus; hardness was increasing with indentation depth. Also, here, increasing hardness can be attributed to changes in the polymer matrix structure through the depth and, in the case of filled materials, the thickness of the PP layer and different crystalline structures for materials based on PP material.

In the case of unrecycled materials with 0, 5, and 10% wt. it might be concluded that low WF concentrations did not affect the material’s hardness since the hardness of the materials with 5 and 10% addition of WF was similar to the hardness of pure PP material, and it stayed the same throughout the indentation depth.

At higher concentrations 20, 30, and 40% wt., a more significant effect of indentation depth on hardness can be observed. The hardness changed by 22% from the first surface layers (750 nm) down to 3500 nm for 40% filled unrecycled material (WF-PP-40) and by 41% for 40% filled recycled material (WF-R-PP-40). This compares to only 5% change for 5% filled unrecycled material (WF-PP-5) and 16% for 5% filled recycled material (WF-R-PP-5).

Comparing the hardness of unrecycled and recycled materials shows that the surface of unrecycled material is harder. As we stated for the modulus, the reason for the lower hardness of recycled material could be the effect of the recycling process and material degradation and the presence of (softer) PE material inside the R-PP. Also, DSC results showed [27] hat materials with PP matrix tend to form structure with higher crystallinity than R-PP, resulting in higher hardness of PP materials.

**Figure 15:**
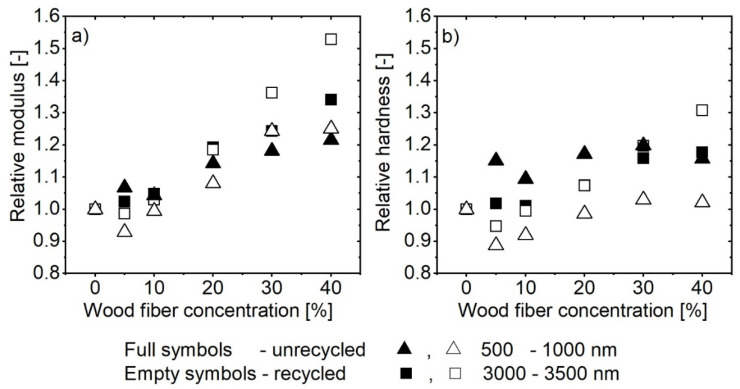
Normalized nanoindentation (**a**) modulus and (**b**) hardness at two selected nanoindentation depths for unrecycled and recycled material as a function of WF concentration.

A further investigation of surface mechanical properties was performed in the two areas marked in Figure 14 with red dashed lines. Specifically, between 500–1000 nm and 3000–3500 nm. The first area was selected since it was the most upper area of the sample, while the second area was selected as an area where properties start to become constant with depth. The results at selected depths are shown in Figure 15. Figure 15a show the relative modulus and (b) relative hardness at selected depths. The values were normalized to the values of pure matrix polymer material (PP and R-PP). By normalizing the values of modulus and hardness to the values of the neat polymer matrix, we obtained relative values of both physical properties, thus eliminating the inherent differences between the two materials.

The relative modulus at the upper sample layers (500–1000 nm), marked with triangles (Figure 15a), is nearly constant up to 10% WF concentrations, as it changes for less than 10%. From 20% on, the modulus starts to increase gradually. At 40 wt.%, the modulus of recycled and unrecycled material is about 25% higher than the pure polymeric matrix material. There is no significant difference between the behavior of recycled or unrecycled material at this layer (500–1000 nm). This could be explained by the comparable thickness of the PP skin layer as shown by the optical microscopy and indicate the material’s homogeneous structure.

From the relative change of hardness (Figure 15b) the hardness of unrecycled material changed by about 15% and stayed constant regardless of the WF concentrations. In contrast, the recycled PP after an initial drop at 5% and 10% wt% did not change significantly compared to pure R-PP material. It appears that the addition of WF makes the surface of unrecycled material harder, while it has a marginal effect in the case of recycled material.

Deeper in the sample, 3000–3500 nm (rectangles), we may again separate the behavior of the material to that at low WF concentrations (up to 10%), where no significant changes of properties could be observed (modulus or hardness). While at higher WF concentrations, a substantial change of modulus and hardness can be seen compared to the neat matrix material’s properties. A more significant change of both properties can be seen in the case of recycled material, where modulus changes by about 50% and hardness by 30% at the highest concentrations, compared to 35% chance of modulus and 18% of hardness in the case of unrecycled material.

Overall, we may conclude that up to 10% WF concentrations there is no significant change to measured mechanical properties. The PP skin layer dominates the mechanical behavior of materials. The observed increase of mechanical properties at lower WF concentrations could be related to the gradual decrease of the PP skin layer. This amplifies the effect of the bottom layers (substrate effect). When the PP skin layer is smaller at higher WF concentrations, the presence of wood fibers reinforces the matrix material, as seen by increased modulus and hardness. The effect of added WF on modulus and hardness, at least at 30 and 40 wt% is not the same for unrecycled and recycled matrix. The higher relative modulus and hardness at of recycled material show that the WF have a stronger reinforcing effect on the overall mechanical properties than unrecycled material.

WF-PP and WF-R-PP composite can be used as a multifunctional material that exploits many properties related to of unique surface-skin-core orientation, surface zeta potential, and increased mechanical strength. On the other hand, the antioxidant properties of these WF-PP/WF-R-PP composites give them a value-added plastic product. Antioxidativity is defined as the ability to inhibit the oxidation process of a free radical. For many applications, the antioxidant properties of filled polymers such as WF-PP/WF-R-PP composite materials are obviously dependent on the amount of filler and they are very much related to the distribution and orientation in the polymer matrix [51,52]. The antioxidant activity was evaluated by DPPH° radical scavenging test and the reducing power test [31,33]. An antioxidant can be broadly defined as any substance that delays or inhibits oxidative damage to a target molecule. The main characteristic of an antioxidant material is its ability to scavenge free radicals and directly exhibit antioxidant activity. Specifically, antioxidant active packaging aims to prevent or slow down the oxidation of certain food components, such as lipids and proteins, resulting in a deterioration of the physical properties (such as taste and color) of these foods. This active material approach requires the intentional incorporation of antioxidants into the packaging materials and their further migration into these food products. On the other hand, the antioxidant surface also prevents the aging of plastics and is therefore very welcome.

**Figure 16:**
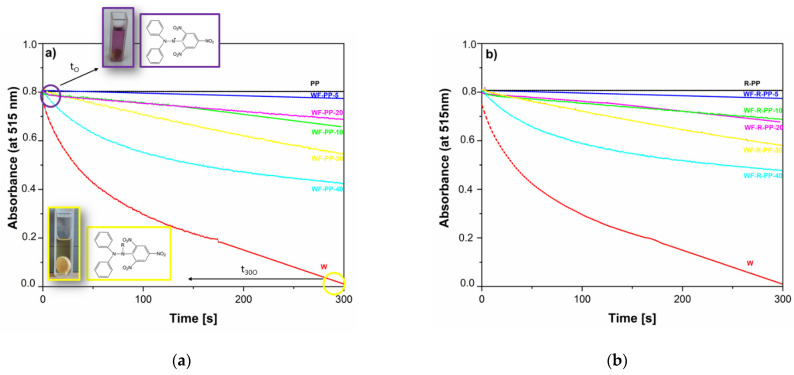
The absorbance as a function of time of (**a**) WF-PP composites and (**b**) WF-R-PP composites.

The antioxidant properties of the composites WF-PP/WF-R-PP can be seen from the color change of the DPPH• radical, which shifts from purple to yellow. Figure 16 represents the evolution of the UV-vis spectra of the WF-PP(Figure 16a)/WF-R-PP(Figure 16b) composites immersed in DPPH• solution and measured at 515 nm and evaluated from 0 to 300 min. As can be seen from the Figure 16. the WF themselves have the higher antioxidant activity which increases with time. PP itself shows the lowest antioxidant activity with no changes in the time interval. Moreover, with the increase of WF addition, the antioxidant activity also increases. In all the cases (5%, 10%, 20%, 30% and 40%), the antioxidant activity is time dependent and increases with the increase in time from 0 to 300 s. However, a plateau value is not observed, and it is assumed that when the time exceeds 300 s, the activity increases even further. The percentage antioxidant activity (AA_100_, AA_200_, AA_300_) at time points 100, 200 and 300 s are shown in Table 3. The results show that the antioxidant activity of WF-PP and WF-R-PP composites increase with increasing WF loading, which is expected since wood is an antioxidant component. Again, the best antioxidant activity is obtained at 40% wood loading, where we also showed the maximum surface availability.

**Figure 17:**
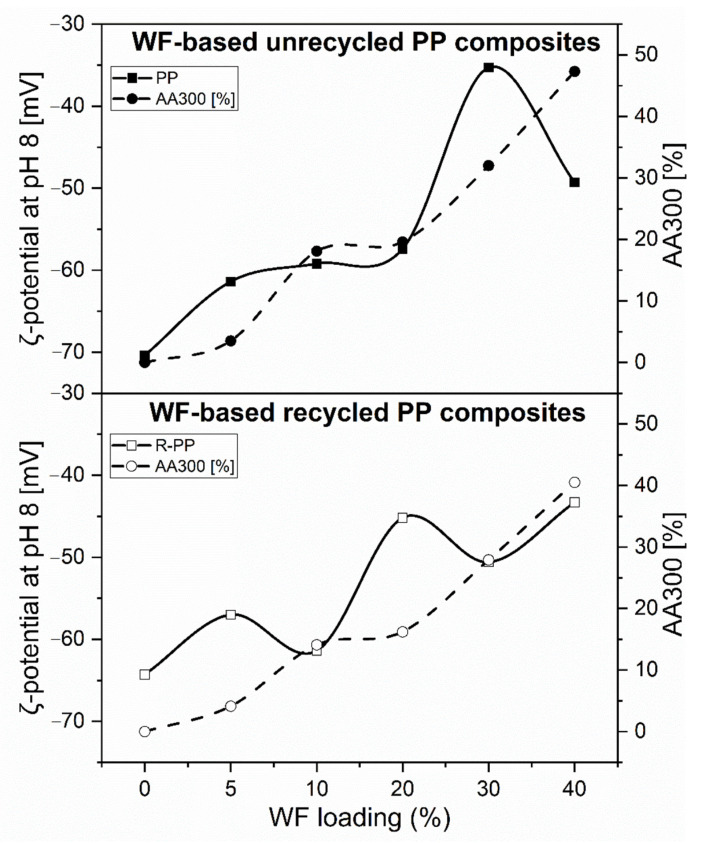
The correlation between negative zeta potential and an antioxidant activity of WF-PP/WF-R-PP composites.

These results indicate the ability of the composites WF–PP/WF-R-PP to act as antioxidants. As the WF load increases, the antioxidant ability of these materials increases. Therefore, we assume that the antioxidant ability of WF–PP composites depend on the active WF groups. It is believed that when the spherical disk of WF–PP is immersed in DPPH-solution, the antioxidant active groups of WF–PP/WF-R-PP deprotonate and donate electrons to the DPPH and finally inhibit the free radical [52]. This method, as a wet method, correlates quite well with another wet “zeta potential method”. It shows a correlation between negative zeta potential as an indication of the hydrophilic character of the surface and antioxidant activity (Figure 17).

It can be concluded that the protonation and wettability that occurred in the water on contact with the material are the driving force for the antioxidant activity. In general, the antioxidant activity of WF-PP and WF-R-PP composites is not preferentially related to WF surface distribution and orientation.

## 4. Conclusions

The plastics industry expects much from advanced materials, but the relatively few that are commercially available cannot meet all applications and expectations. In this context, hybrid thermoplastic materials with renewable fillers can potentially provide all the benefits of traditional filled plastic composites and avoid their environmental drawbacks, such as non-biodegradability and extreme pollution. The influence of WF loading and recycling on the surface properties of wood-polymer composites was investigated. In the diffraction patterns of all WF–PP/WF-R-PP composite samples, the α-monoclinic PP formations were preferentially present. In general, the relationship between WF loading to PP/R-PP with increased distribution of the well-known phenomena of skin layer thickness of WF–PP/WF-R-PP composites was highlighted. Moreover, the ATR-FTIR measurements showed that up to 30% of the addition of WF to the PP, WF–PP composites accelerated the surface functional groups present in the wood. Indeed, the increased loading of WF in PP resulted in a thicker layer of the upper skin layer of WF–PP/R-PP polymer composites, as also observed with optical microscopy. WF concentration also caused changes in mechanical properties (nanoindentation modulus and hardness). Two distinct ranges were observed; at low concentrations (up to 10%), no significant changes in properties were observed. At concentrations above 20%, significant changes were observed (up to 40%). This behavior can be associated with the WF density and the formation of a particle network when the WF concentration is increased, as well as with the formation of a different crystalline structure in the case of non-recycled PP. Furthermore, we have shown that the properties in the top surface layers of the samples were similar to the properties of the pure matrix material at low concentrations and increased slightly at high concentrations. This indicates that a layer of pure polymer matrix material formed on the upper surface of the sample; however, with increasing WF concentration, this layer appears to decrease. At sufficiently high WF concentration, the fibers prevented the formation of a pure polymer layer on the surface, which was also confirmed by ATR measurements where the characteristic peaks for WF functional groups were found on the surface layers of the materials with 30, 40% WF concentration. The zeta potential measurements showed an increased surface hydrophilicity with the introduction of more polar groups of WF on the surfaces of the WF composites as directly responsible for the increased antioxidant activity, which increases with the added amount of WF. The nanoindentation modulus and hardness also increase with the added amount of WF, which is significant at 30% and 40% of WF, respectively.

As mentioned above, many such materials act on the interphase (packaging materials, containers, bins.), so the first mechanical stresses occur on its surface, and it is therefore extremely important to improve it. Moreover, with introduced hydrophilicity and antioxidant activity packaging applications can be seen where anti-fog, properties are essential to prevent condensation and antioxidant activity prevents oxidation processes in the packaging system. All these properties inhibit the perishability of packaged goods and prolong their life. In addition, antioxidant activity influences the slower aging of plastics. It was shown that important surface properties could be improved by the addition of wood fillers without further adhesion chemicals. Zeta potential measurements also show that all materials still have anionic character, indicating adsorption affinity to cationic substances and their electrostatic interactions. The latter is extremely important in the surface functionalization of these composites, such as antimicrobial agents, where most antimicrobial agents have cationic character.

## Figures and Tables

**Table 1:** Prepared materials composites and names of individual materials.

Wood Fiber Loading [wt.%]	0	5	10	20	30	40
Unrecycled PP	PP	WF-PP-5	WF-PP-10	WF-PP-20	WF-PP-30	WF-PP-40
Recycled PP	R-PP	WF-R-PP-5	WF-R-PP-10	WF-R-PP-20	WF-R-PP-30	WF-R-PP-40

**Table 2:** ζ-potential for PP and R-PP-softwood fiber composites and deviation between measured and predicted results (assuming a weighted average of the zeta potential for PP and R-PP and spruce, respectively).

Composites Material	ζ-Potential at pH 8 (mV)	ζ-Potential at pH 8 (mV)
WF-PP-0	−70.4	n/a
WF-PP-5	−61.4	+5.8
WF-PP-10	−59.2	+4.7
WF-PP-20	−57.4	+9.0
WF-PP-30	−35.3	+15.6
WF-PP-40	−49.3	−4.9
WF-R-PP-0	−64.3	n/a
WF-R-PP-5	−57.0	+4.4
WF-R-PP-10	−61.4	−2.9
WF-R-PP-20	−45.2	+7.4
WF-R-PP-30	−50.6	−4.9
WF-R-PP-30	−43.3	−2.5

**Table 3:** Antioxidant activities of WF-PP and WF-R-PP composites represented by the percental decrease of absorbance of DPPH at 515 nm after 100, 200 and 300 s.

Composite’s Material	AA_100_ (%)	AA_200_ (%)	AA_300_ (%)
PP	0	0	0
WF-PP-5	0.99	2.11	3.5
WF-PP-10	5.60	11.8	18.1
WF-PP-20	7.34	13.7	19.6
WF-PP-30	11.9	22.9	32.0
WF-PP-40	31.5	41.5	47.3
WF	63.1	81.5	98.6
R-PP	0	0	0
WF-R-PP-5	0.74	2.11	4.11
WF-R-PP-10	5.97	9.96	14.1
WF-R-PP-20	5.10	9.96	16.2
WF-R-PP-30	10.2	19.3	27.9
WF-R-PP-30	26.6	35.7	40.5
WF	63.1	81.5	98.6

## Data Availability

Data is contained within the article.
